# A Comparative Analysis of Sector Classification and the Impacted Canine Grading System by Kumar and Daigavane in Patients With Unilateral Cleft Lip and Palate Utilizing Orthopantomogram (OPG) as a Diagnostic Tool

**DOI:** 10.7759/cureus.67079

**Published:** 2024-08-17

**Authors:** Nikita Soni, Priyanka Niranjane, Pallavi Daigavane, Ranjit Kamble

**Affiliations:** 1 Department of Orthodontics and Dentofacial Orthopaedics, Sharad Pawar Dental College and Hospital, Datta Meghe Institute of Higher Education and Research, Wardha, IND

**Keywords:** impacted canine, grading system, kumar and diagavane classification, sector classification, cleft lip and palate (clp), impacted maxillary canine

## Abstract

Introduction

Non-syndromic oral clefts, affecting one in 700 newborns in India, are the most prevalent craniofacial anomalies, with genetic or environmental causes impacting various life aspects. Studies indicate higher dental disturbances, particularly impacted canines, in cleft lip and palate (CLP) patients compared to non-cleft individuals. Impacted canines, trapped by hard tissues, require early diagnosis to prevent orthodontic issues. The widely used Ericson and Kurol method employs orthopantomograms (OPGs) to classify canine impaction in typical children. However, diagnosing canines in CLP patients is challenging due to palate defects and post-grafting complications. This study aims to compare the utility of the Kumar and Daigavane (KD) grading system and the sector classification to determine the best method for diagnosing impacted canine eruption paths.

Method

This cross-sectional comparative observational study was conducted at Sharad Pawar Dental College's Department of Orthodontics and Dentofacial Orthopaedics. The sample size, calculated using a significance level of 5% and a prevalence of 1%, required a minimum of 16 participants aged 9-11 years with non-syndromic clefts and impacted canines. Patients with systemic diseases or over 12 years of age were excluded. The sectoral and KD classification systems collected and evaluated OPGs from qualifying cleft patients. Sector classification considered the angle between the occlusal plane or canine tip and the adjacent tooth's long axis, while KD's classification considered the Frankfort horizontal plane, occlusal plane, vertical height from the occlusal plane, canine apex root position, and canine exposure to the cleft defect.

Results

The study found an 81.25% agreement between the KD grading system and the sector classification, with a Cohen's kappa value of 0.586, indicating a moderate agreement. The KD system showed 81.82% sensitivity and 80.00% specificity, with positive and negative predictive values of 90.00% and 66.67%, respectively. The receiver operating characteristic (ROC) curve analysis revealed the KD system's superior performance in identifying impacted and non-impacted canines compared to the sector classification.

Conclusion

The KD grading system demonstrated higher efficacy than the sector classification for evaluating the impacted canines in children with unilateral cleft lip and palate (UCLP). The KD system's high sensitivity and specificity make it a valuable tool for predicting canine eruption paths and addressing anatomical challenges in cleft conditions. This study highlights the need for accurate diagnostic tools tailored to cleft patients and contributes to advancing orthodontic treatment outcomes through improved classification systems.

## Introduction

Non-syndromic oral clefts, which affect one in 700 newborns in India, are the most prevalent craniofacial structural anomaly. These clefts can be genetic or environmentally induced, impacting various facets of an individual's life [1]. Studies have shown that cleft lip and palate (CLP) patients have significantly more dental disturbances, particularly impacted canines, than non-cleft patients [2]. An impacted canine is malpositioned, surrounded by hard tissues, and thus trapped or immovable, preventing it from emerging into the oral cavity. Adrian Becker identifies multiple factors for maxillary canine impaction, including local complex tissue obstructions, pathologies like supernumerary teeth, developmental disturbances, and hereditary factors linked to lateral incisor anomalies [3]. Early diagnosis of impacted canines can prevent associated problems affecting other teeth. Diagnosing and correcting impacted canines will avoid future orthodontic difficulties, especially since canines have the most extended developmental period and a long path to eruption, which may be attributed to alveolar grafting, which seals the oronasal fistula in CLP patients [4]. Several diagnostic approaches are available for evaluating the progression of canine eruptions. Ericson and Kurol provide the most widely used methods for determining the position of an impacted canine using a two-dimensional image known as an orthopantomogram in typical children aged 9 to 12 years. They classified five types of canine impaction based on the angle formed by the central incisor, lateral incisor, and canine's long axes. Determining the location and eruption path of canines in cases of the cleft is challenging due to defects in the palate or issues following alveolar bone grafting [5]. Additionally, cleft patients face a higher incidence of canine impaction after alveolar bone grafting than non-cleft patients due to differences in arch length, tooth trauma during root development, or incomplete canine development [6]. A new classification system known as the Kumar and Daigavane (KD) grading system has been developed to evaluate the position of impacted canines or canine eruption pathways in patients with CLP. According to this system, the position of the canine is classified into four grades and the prognosis is determined [7]. This study evaluates how the KD classification system, specifically designed for patients with CLP, compares with the sector classification system, which does not account for cleft conditions. The KD system considers the unique anatomical and developmental issues associated with CLP, such as changes in bone structure and impacts from previous surgeries, potentially offering a more precise assessment of canine impaction. By contrasting these two systems, the study aims to see if the KD classification provides a meaningful improvement in diagnosing and managing impacted canines in cleft patients compared to the more general sector system. This comparison is essential for enhancing diagnostic accuracy and optimizing treatment strategies, leading to better outcomes for individuals with clefts. Further investigation is required to determine the effectiveness of the sector classification in these specific patients. The present study has been planned to evaluate the applicability and efficacy of this new KD grading system in the already widely used sector classification system for assessing and predicting the position of canines in CLP cases. The aim is to compare the utility of the KD grading system and the sector classification of impacted canine classification methods to determine which method holds the most significant prognosis for diagnosing impacted canine eruption paths.

Objectives

The objectives are to evaluate the suitability of the KD grading system and the sector classification for assessing canine position in CLP cases using 2D orthopantomograms and to compare their effectiveness in predicting canine emergence path in non-syndromic cleft patients.

## Materials and methods

**Figure 1:**
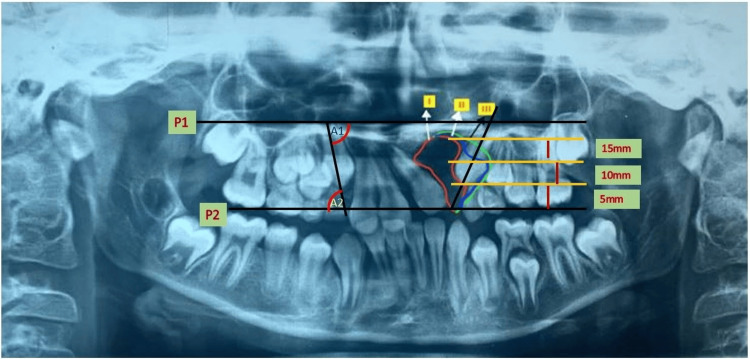
The Kumar and Daigavane (KD) classification for impacted canine done on cleft orthopantomogram (OPG).

This cross-sectional comparative observational study was conducted in the Department of Orthodontics and Dentofacial Orthopaedics in collaboration with the Department of Oral Medicine and Radiology. The purpose of the study was explained to the study participants, their consent was obtained, and patient confidentiality was maintained throughout the study. Eligibility criteria included patients with impacted canines, aged 9-11 years, who were non-syndromic cleft patients without systemic diseases. Patients with erupted canines, those over 12 years of age, syndromic patients, and those with systemic diseases and incomplete clinical records were excluded. Data collection involved obtaining orthopantomograms (OPGs) from cleft patients meeting the inclusion criteria. Maxillary-impacted canines were evaluated using both the sector classification system and KD's classification system. The sector classification considered the angle between the occlusal plane or canine tip and the long axis of the adjacent tooth. In KD's classification, the Frankfurt horizontal plane (P1) and occlusion planes (P2) were used to assess the canine's angulation, measure the vertical height from the occlusion plane to the cusp tip, and determine the root apex position about the canine. Additionally, canine exposure within the cleft defect area was categorized into three grades [7] (Figure 1).

The sensitivity and specificity of the sector classification and the Impacted Canine Grading System by Kumar and Daigavane were compared to diagnose canine impaction in patients with unilateral cleft lip and palate using orthopantomogram (OPGs). OPG images were independently assessed using both grading systems, and results were compared and established through clinical examination, surgical findings, and consensus by the orthodontists (Table 1).

**Table 1:** Kumar and Daigavane’s impacted canine grading system for cleft lip and palate.

	Grade I	Grade II	Grade III	Grade IV
Clinical finding	Presence of a labial or palatal canine bulge at the alveolar process	Presence of a labial canine bulge above the alveolar process and till the depth of the vestibule and in the palatal region palatal bulge is seen in between the rugae area	Presence of a labial bulge above the depth of the vestibule and the palatal bulge beyond the rugae area	Absence of any labial or palatal canine bulge
Angulation measured from the Frankfurt horizontal plane (A1) and occlusion plane (A2)	90^0^ ± 10^0^	45^0^ ± 10^0^	180^0^ ± 10^0^ and above	Distally angulated
Vertical height measured from occlusion plane	5 ± 2 mm	10 ± 2 mm	15 ± 2 mm	Above 15 mm
Apex location	Above canine position	Above first premolar	Above second premolar	Towards lateral incisor
Exposure to cleft defect	Bone lining at the tooth	Only crown in the defect area	Crown and half root in the defect area	Only one-fourth apex in the bone
Inference	Good prognosis for canine eruption and alignment	Average prognosis for canine eruption and alignment	Poor prognosis for canine eruption alignment	Hopeless prognosis for canine eruption alignment

Sample Size Calculation

The sample size of 16 (each in both the groups) was calculated using the formula based on the prevalence of cleft lip and palate 1%:

$n = \frac{Z_{1-\alpha/2}^2 \cdot p \cdot (1-p)}{D^2}$

with $Z_{1-\alpha/2} = 1.96$ at a 5% level of significance where P = prevalence of cleft lip and palate 1% (0.01), D = estimated error (0.05%), and N = population size [8].

Statistical Analysis 

Statistical analysis was performed using SPSS for Statistics version 23 (IBM Corp., Armonk, NY). Descriptive statistics tests were conducted for mean distribution and standard deviation. Cohen's kappa value assessed the agreement between the two systems, and the receiver operating characteristic (ROC) curve was used to compare diagnostic systems for cleft lip and palate patients. The sensitivity and specificity were calculated to determine the true positives, true negatives, false positives, and false negatives. SPSS statistical software reported these metrics with 95% confidence intervals.

## Results

The agreement between the sector classification and the KD classification for diagnosing canine impaction in patients with unilateral cleft lip and palate (UCLP) was 81.25%, with a Cohen's kappa value of 0.586, indicating a moderate level of agreement. In our analysis of 16 patients, both systems were correctly identified in nine cases of canine impaction and in four cases impaction was not present. However, there were discrepancies: the sector classification incorrectly identified two patients as having canine impaction, which the KD classification accurately identified them as non-impacted. The KD classification incorrectly classified one patient, which was correctly identified by the sector system. The results indicate that there is no statistically significant difference between the two systems in terms of diagnostic accuracy, suggesting that both classification methods are comparably effective (Table 2).

**Table 2:** Comparative analysis of sector classification and Kumar and Daigavane (KD) classification for diagnosing canine impaction with Cohen's kappa statistics.

		Sector classification		
		Present	Absent	Total
KD classification	Present	9	1	10
	Absent	2	4	6
	Total	11	5	16

Table 3 provides statistical measures for a diagnostic test, presenting its sensitivity, specificity, positive predictive value (PPV), negative predictive value (NPV), and overall accuracy, along with their respective 95% confidence intervals (CI). The test's sensitivity is 81.82%, with a confidence interval ranging from 48.22% to 97.72%, indicating its ability to identify those with the condition correctly. The specificity stands at 80.00%, with a confidence interval (CI) from 28.36% to 99.49%, reflecting the test's capability to identify those without the condition correctly. The positive predictive value is 90.00%, with a CI of 60.40% to 98.15%, suggesting a high likelihood that a positive test result accurately indicates the presence of the condition. The negative predictive value is 66.67%, with a CI ranging from 34.64% to 88.30%, showing the probability that a negative test result correctly predicts the absence of the condition. Lastly, the accuracy of the test is 81.25%, with a CI between 54.35% and 95.95%, representing the proportion of actual results (both positive and negative) among all test outcomes (Table 3).

**Table 3:** Accuracy metrics for Kumar and Daigavane's (KD) canine classification system.

Statistic	Value (%)	95% CI
Sensitivity	81.82	48.22-97.72%
Specificity	80.00	28.36-99.49%
Positive Predictive Value (*)	90.00	60.40-98.15%
Negative Predictive Value (*)	66.67	34.64-88.30%
Accuracy (*)	81.25	54.35-95.95%

**Figure 2:**
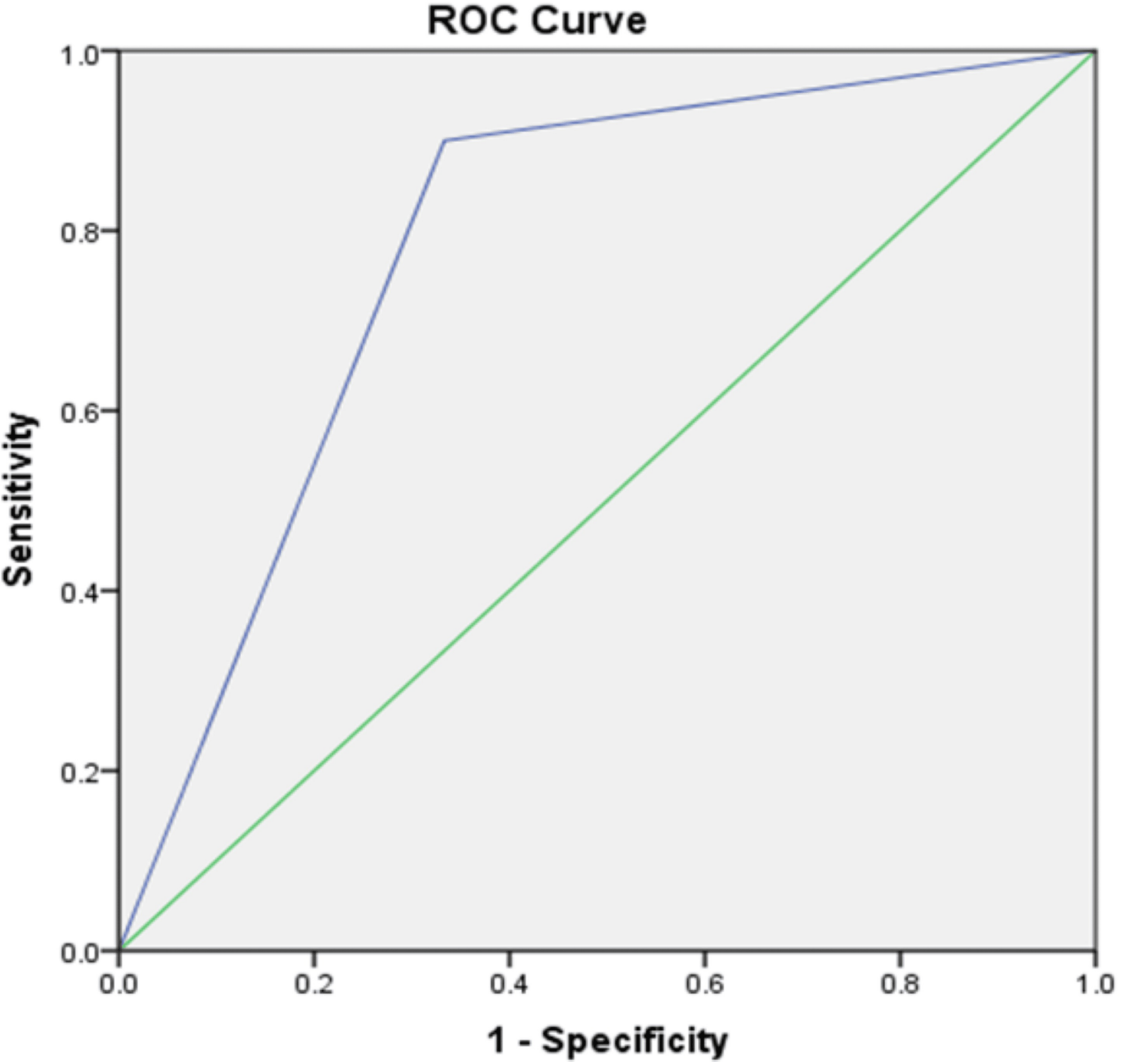
ROC Curves comparing the Kumar and Daigavane (KD) classification (blue line) with sector classification (green line) in cleft lip and palate.

The ROC curve analysis shows that KD system outperforms the sector classification. The sharp increase of the blue line towards the top-left corner indicates excellent sensitivity and specificity, reflecting a solid ability to identify impacted and non-impacted canines accurately. Although not explicitly shown, the area under the blue ROC curve indicates superior performance (Figure 2).

## Discussion

This study evaluates the KD grading system against the sector classification for infants with unilateral cleft lip and palate (UCLP) using orthopantomograms (OPGs), offering valuable insights into dental anomalies in cleft patients. The accurate tracking of impacted canines' eruption paths is essential for effective orthodontic planning, and this research aims to identify the most effective method. Kumar and Daigavane's system, designed specifically for cleft patients, shows superior diagnostic performance with higher sensitivity and specificity, addressing the unique anatomical challenges of cleft conditions. The significance of this study lies in its development of a grading system to predict the prognosis of impacted canines in cleft patients, as traditional sector classification may be inadequate due to bony defects or complications. The KD classification system is user-friendly, integrates easily into routine practice with standard OPGs, and provides precise assessments tailored to cleft lip and palate patients. It enhances treatment planning, facilitates more transparent communication among dental professionals, adapts to various imaging techniques, and improves predictive accuracy for canine eruption and alignment, supporting more effective interventions. Comparative studies, such as those by C. Weismann et al. [8], highlight a higher rate of canine displacement in craniofacial disorder patients than controls, underscoring the need for early diagnostic interventions. Sara Rizell et al. [9] found significant differences in the positioning of impacted canines between cleft and non-cleft sides, emphasizing the limitations of traditional panoramic radiographs due to dental anomalies and timing of bone grafting. Deep Shah et al. [10] stress the importance of radiographic techniques in diagnosing and planning treatment for impacted canines, noting that while OPGs offer a general view, cone beam CT (CBCT) provides more precise localization and assessment of root resorption and adjacent bone pathologies. Korde et al. [11] measured angulation and position to guide surgical approaches for impacted canines, highlighting the need for prognosis evaluation using sector categorization to enhance treatment outcomes. Russell et al. [12] discussed the impact of secondary alveolar bone grafting on canine eruption, noting that proper detection and timely intervention are crucial for patients with orofacial clefts. This study compares the Kumar and Daigavane grading system with the traditional sector classification for assessing impacted CLP patients using OPGs. While the sector classification system is commonly used for non-cleft cases, it falls short for CLP patients due to its reliance on anatomical features often altered or missing in cleft conditions, such as the continuous alveolar ridge and lateral incisors. Kumar and Daigavane's system, designed specifically for these unique challenges, provides superior diagnostic performance with enhanced sensitivity and specificity, addressing the limitations of conventional methods [7]. This comparison highlights the need for tailored diagnostic tools that improve orthodontic treatment planning for CLP patients, ensuring more accurate and effective management of impacted canines. The study confirms that while KD's grading system could be further refined, it is suitable for clinical assessments of impacted maxillary canines in UCLP patients. It advances orthodontic treatment by providing a more effective classification system for managing this population's impacted canines and anatomical challenges. Future improvements include validating the KD system with more prominent, diverse patient groups, comparing its effectiveness with emerging classification systems, and integrating advanced imaging technologies. Feedback from a broader range of dental professionals could also refine the system's usability and adaptability in various clinical settings. These steps will enhance the system's diagnostic capabilities and improve treatment planning for impacted canines in cleft lip and palate patients. The strengths of this study lie in its innovative approach to evaluating impacted canines in UCLP patients. The KD grading system, designed explicitly for cleft patients, demonstrated high sensitivity and specificity, making it a potentially superior tool for predicting the eruption path of canines compared to the traditional sector classification. The study addresses a significant gap in the existing diagnostic methods, offering a tailored solution that considers the unique anatomical challenges presented by cleft conditions. Furthermore, the rigorous statistical analysis and the comparison of two well-defined grading systems add credibility to the findings, paving the way for improved clinical outcomes in orthodontic treatment planning for cleft patients. This study, while providing valuable insights, has its limitations. Firstly, the sample size was relatively small, which may affect the generalizability of the findings. Additionally, the reliance on 2D imaging (orthopantomograms) may have introduced inaccuracies in assessing the exact position of impacted canines, as it cannot show bucco-palatal locations. The study also focused solely on non-syndromic cleft lip and palate (UCLP) patients, which may limit the applicability of the results to a broader population, including those with other craniofacial anomalies. Finally, while the KD grading system showed promise, the study needed to explore its application with advanced imaging techniques like CBCT, which could provide a more comprehensive evaluation.

## Conclusions

This research demonstrated the effectiveness of Kumar and Daigavane's grading system in evaluating impacted canines in children with unilateral cleft lip and palate (UCLP). The findings revealed that this new system outperforms the traditional sector classification by offering higher sensitivity and specificity. The new grading system is particularly significant for patients with cleft conditions, who face unique anatomical challenges such as bony defects in the palate and complications from post-alveolar bone grafting. Kumar and Daigavane's system enhances the prediction of canine eruption paths, leading to more precise orthodontic treatment planning and improved patient outcomes. The study highlights the need for a new diagnostic grading system tailored to impacted canines in cleft conditions, as current methods may not adequately address the complexities of these cases. Thus, it contributes substantially to orthodontics by offering a structured approach and protocol for better managing impacted canines in cleft patients and elevating the standard of care.
